# Brain stimulation for treatment and enhancement in children: an ethical analysis

**DOI:** 10.3389/fnhum.2014.00953

**Published:** 2014-12-18

**Authors:** Hannah Maslen, Brian D. Earp, Roi Cohen Kadosh, Julian Savulescu

**Affiliations:** Oxford Uehiro Centre for Practical Ethics and Oxford Martin School, University of OxfordOxford, UK

**Keywords:** brain stimulation, pediatric ethics, cogntive enhancement, functional trade-offs, autonomy

## Abstract

Davis (2014) called for “extreme caution” in the use of non-invasive brain stimulation (NIBS) to treat neurological disorders in children, due to gaps in scientific knowledge. We are sympathetic to his position. However, we must also address the *ethical* implications of applying this technology to minors. Compensatory trade-offs associated with NIBS present a challenge to its use in children, insofar as these trade-offs have the effect of limiting the child’s future options. The distinction between treatment and enhancement has some normative force here. As the intervention moves away from being a treatment toward being an enhancement—and thus toward a more uncertain weighing of the benefits, risks, and costs—considerations of the child’s best interests (as judged by the parents) diminish, and the need to protect the child’s (future) autonomy looms larger. NIBS for enhancement involving trade-offs should therefore be delayed, if possible, until the child reaches a state of maturity and can make an informed, personal decision. NIBS for treatment, by contrast, is permissible insofar as it can be shown to be at least as safe and effective as currently approved treatments, which are themselves justified on a best interests standard.

Davis (2014) has called for “extreme caution” in the use of non-invasive brain stimulation (NIBS) methods to treat neurological disorders in children. His focus is on transcranial magnetic stimulation (TMS) and transcranial direct current stimulation (tDCS), which, respectively, involve passing either an electro-magnetic field or a small direct current through the skull in order to modulate neuronal activity. To justify his position, Davis calls attention to four major issues, framed as “known unknowns” in the current literature:

1. unknown effects of brain stimulation, and unknown mechanisms for producing those effects;
2. unknown side-effects of stimulation (both short- and long-term);
3. a lack of clear dosing guidelines; and
4. a lack of translational studies from adults to children.

As Davis rightly points out, “children [cannot] be considered as ‘small adults’ when testing medical interventions” (p. 2). This is especially the case for interventions into the central nervous system, since a child’s developing brain may respond differently to stimulation compared to that of an adult. Indeed, research shows that the brain continues to develop even after the age of majority (Sowell et al., 2003). Nevertheless, Davis balances his plea for caution with longer-term optimism. He argues that—when used with care—brain stimulation in children does appear to be safe and well-tolerated, and may even turn out to be “associated with fewer and less unpleasant side-effects than the neuroactive drugs [such stimulation is] intended to replace” (p. 3).^1^

We are sympathetic with Davis’ argument (Cohen Kadosh et al., 2012). Put simply, caution and sustained clinical scrutiny are required, both for research into the effects of pediatric brain stimulation and for the application of such technology. Yet while further empirical studies into appropriate dosing, side-effects, and so on should allow for brain stimulation in children to be made generally safer (as well as more effective therapeutically), we must also address the gaps in our understanding of the *ethical* implications of applying this technology to minors.

In this article, we aim to contribute to such an understanding. To frame our discussion, we draw a distinction between the use of NIBS (but see Davis and van Koningsbruggen, 2013)^2^ as a form of *treatment* for a recognized neurological disorder, and its use as a form of *enhancement* in healthy children. Although we have argued in previous work that the treatment/enhancement distinction tends to break down in the case of adults (see Earp et al., 2014), in the case of children, we suggest, it has greater normative force. This is because, we argue, the relative weights of (parental judgements of) beneficence vs. respect for autonomy *shift* as the decision pertains more to “enhancement” than to “treatment”.^3^^,^^4^

The tension between these two factors arises because some interventions may involve compensatory trade-offs or functional losses, such as potential cognitive costs in the case of brain stimulation. When these trade-offs have the effect of limiting the child’s future options, they pose a threat to his or her (future) autonomy. Whilst choosing to “treat” a child will sometimes be in his or her best interests *even if* it precipitates cognitive trade-offs, interventions intended to “enhance” may not be justified in this way. In the absence of a clear pathology, we suggest, greater relative weight should be placed on the child’s (future) autonomy, at least in part because the certainty with which the parents can determine what would be in his or her best interests is likely to be significantly reduced.

Given this, we argue that brain stimulation for “enhance­ment”—insofar as it involves a more controversial weighting of benefits vs. risks and costs—should be delayed until the child has reached a state of maturity. In this way, she can make an informed, personal decision about the proposed intervention. Brain stimulation for “treatment”, by contrast, is permissible insofar it can be shown to be at least as safe and effective as currently approved treatments (which are themselves justified on a best interests standard).

## The permissibility of treating neurological disorders in children

To begin our discussion, we ask, what makes pediatric “treatment” permissible in general? By “treatment” we intend to call to mind such interventions as surgery to correct a heart defect, or the administration of antibiotics to address an infection. In these cases, a disease or deformity is present that threatens the child’s well-being, and the treatment is the best available means (or a good-enough means) to mitigate that threat. Thus, although (a) the child cannot strictly consent to the intervention, (b) the intervention may carry considerable risk, and (c) it may involve even a gross intrusion into the child’s bodily sphere, it is nevertheless considered to be morally permissible. Such an intervention is permissible because, and insofar as, it is in the child’s best interests—all things considered (see Hope et al., 2008).

We can extend this reasoning to the case of brain stimulation. If a child is experiencing significant psychological and/or physical burdens due to a neurological disorder, the benefits of treatment with stimulation might very well be in the child’s best interests in the sense just described. In fact, this could turn out to be the case *even if* some significant negative side-effects were generated, so long as the overall costs to the child (including the cost to autonomy) were outweighed by the benefits of performing the stimulation before an age of consent. On these grounds, it could be considered permissible, assuming that it were shown to be at least as safe and effective as other, more established treatment paradigms.^5^

## Enhancement and the child’s interest in autonomy

What about the case of “enhancement”? Ethicists are divided on the question of whether parental enhancement choices are in the child’s best interests and this is often framed in terms of a consideration of the child’s interest in (future) autonomy, or self-determination. Some have argued that the enhancement of a child might lead her to feel unfree to pursue her own life-projects due to the fact that decisions about her traits and capacities have been chosen for her. In developing this argument, Habermas (2003, p. 50) has argued that, in the case of genetic enhancement (i.e., selecting for specific traits, such as intelligence), the parents’ choices represent intentions and expectations relating to their child’s life. Such expectations, he suggests, lead to the stifling of the child’s freedom to develop in his or her own way.

Others have argued that enhancement technologies would not undermine autonomy, insofar as they increase the options available in an individual’s choice set. For example, Bostrom (2005) claims that an enhanced child might “enjoy significantly *more* choice and autonomy in her life, if the modifications were such as to expand her basic capability set. Being healthy, smarter, having a wide range of talents, or possessing greater powers of self-control are blessings that tend to open more life paths than they block” (p. 212). Such an analysis tends to assume that enhancement has the overall effect of increasing *objective* opportunities, even if a child might *experience* her freedom as being constrained by parental expectations. However, as we will now discuss, in the case of brain stimulation, the assumption of “more choice” may sometimes be mistaken. The arguments we make in what follows are about objective, not subjective, curtailment(s) of freedom.

## Brain stimulation and cognitive trade-offs

While early research into brain stimulation in healthy adults has focused on its potential to enhance cognitive functions, the cognitive costs that might be associated with such enhancement have largely been neglected. However, as Davis points out, no brain region exists in isolation. Indeed, there is evidence that enhancing one aspect of cognition may be detrimental to other cognitive faculties, making neuromodulation “a zero-sum proposition” (Brem et al., 2014; but see Luber, 2014). For example, it has been shown that enhancing cognitive performance on one task can be associated with poorer performance on a different cognitive task (Iuculano and Cohen Kadosh, 2013; Sarkar et al., in press).

It must be acknowledged that the evidence for such enhancement tradeoffs has thus far been obtained only from well-controlled laboratory experiments that have poor ecological validity. However, this preliminary evidence should alert us to the possibility of similar trade-offs that might occur in more ecologically valid settings. Laboratory experiments can help to demonstrate what would be theoretically expected, based on the cognitive function that is targeted and the brain regions that are stimulated. Crucially, such experiments suggest that it is theoretically likely that enhancement of one domain of cognition will sometimes come at the cost of impairment in another. Thus, any decision to enhance could be also a decision to impair. When this is coupled with the emerging probability of long lasting effects on the brain (see Snowball et al., 2013), a situation arises in which parents might inadvertently or even knowingly limit (at least some) future options for their children when they choose to enhance particular capacities at the expense of others.

For example, imagine a parent who has aspirations for her child to be the star of the school’s quiz team. The parent encourages the child to memorize facts whilst her brain is stimulated to enhance long-term memory. However, as a result, the child’s visuospatial working memory is impaired and her ability to quickly solve mental arithmetic problems suffers (see de Jongh et al., 2008 for a review of such trade offs with respect to pharmacological enhancements). Although the child performs well on general knowledge tests, she performs less well in mental arithmetic: mathematics-related pursuits are, to a certain degree, limited as a result of the intervention.

In this example, by choosing to enhance the child’s long-term memory and, correspondingly, the ease with which activities employing this particular cognitive capacity can be pursued, the parent is also choosing to impair a different capacity, making the pursuit of activities involving visuospatial working memory more difficult. It is our contention that making these opportunity-limiting choices on behalf of the child may not be permissible. This is the case even if opportunities associated with the enhanced cognitive domain are increased. Parents cannot know what the child will grow up to value and so should not restrict opportunities based on what *they* want their child to pursue. Whilst there are many decisions that parents can make in the best interests of their child, which cognitive capacities are more valuable, we contend, is usually not one.

This argument applies most strongly to cases in which there is (roughly) a one-to-one trade-off. However, if a given enhancement intervention *substantially* increased function in one domain, while only *slightly* reducing function in another (as judged by a reasonable observer), then the decision would turn more heavily on considerations of what would be in the best interests of the child, overall. Such valuations are hard to make, and are likely to be highly subjective in many cases. The more subjective they are (that is, the less clear an “objective” observer would be about the relative weights to assign to the enhanced vs. diminished capacities), the more the decision about intervening should be left to the individual who must live with the consequences.

## At what age can children decide to be “enhanced”?

Let us summarize our argument so far. First, when “enhancement” interventions involve a functional trade-off, the agent whose relevant capacities will be altered should usually be the one to make a decision about whether the intervention is desirable, all-things-considered. However, young children are unlikely to know which capacities they will value later in life, since their self-knowledge and ability to make and pursue long-term goals is yet to develop. Therefore, there is a problem in terms of a child’s capacity to consent—that is, to fully understand what an intervention involves and what the material consequences will be. Moreover, there is a problem in terms of the child’s limited insight into what she will value over time. At what point, then, can children make meaningful decisions regarding self-enhancement, taking into consideration the apparent risk of cognitive trade-offs?

To begin with, we should point out that a child’s inability to provide informed consent does not make pediatric interventions impermissible *per se*. As we have already suggested, when it comes to treatment, at least, parents (or legal guardians) can legitimately make decisions in the best interests of the child. Similarly, when an intervention is carried out for purposes of medical research, a child’s lack of capacity to consent is not necessarily prohibitive either. In these cases, clinicians or researchers must seek (and obtain) the child’s *assent* to participate in the study (as well meet all other ethical requirements, see Caldwell et al., 2004).^6^

For minor interventions, then—such as venipuncture for the purposes of a study—a child’s assent may be all that is needed. This is because the risks that are associated with such a procedure are either immediate and transitory (e.g., pain, stress, or discomfort) or rare (e.g., hemorrhage or infection), assuming that the intervention is properly performed. By contrast, the effects of brain stimulation for “enhancement” may have consequences that reach far into the child’s future. Therefore, in order to evaluate the reasons one might have for refusing such an “enhancement” (such as a desire to leave one’s cognitive functions intact), one must be capable of meaningful temporal self-projection. Yet such projection is usually not possible for very young children.^7^

It may be possible, however, for older children and/or adolescents. Accordingly, some scholars have suggested that genuine *consent* may be possible before an age of legal majority (typically 18), at least for certain kinds of “medical” interventions (see, e.g., Levy et al., 2003). For simple procedures with minimal risks, children as young as 10 may be capable of giving age-appropriate consent. As the risks increase, however, and as the need for temporal projection becomes more central to the decision-making process, a higher threshold for consent is required. In the case of “enhancement” decisions involving potential trade-offs, such as the impairment of a cognitive capacity, the threshold should be higher still.

This is for two reasons: first, as we have discussed, a child’s brain is still developing, and in numerous ways that are not yet understood. Indeed, even adolescent and adult brains continue to develop. Nevertheless, and second, adolescents (and adults) have much greater insight—compared to very young children—into their own future values. It is this forward-looking capacity, we contend, that is especially important when making decisions about how to weigh the relative value of different cognitive functions; and younger children seem to lack this capacity. Therefore, in the case of pediatric enhancement involving long-term cognitive tradeoffs, we suggest that consent may be (ethically) obtainable by later adolescence, perhaps around the age of 16, but usually not earlier than this.

## Parents’ traditional influence on children’s skill development

A first response to our argument might be to point out that parents already make many (relatively unproblematic) decisions when, for example, they allow their children to take part in certain extra-curricular activities but not others. A parent might encourage her child to go to drama club instead of French lessons or to practice football rather than sing. However, there are important differences between these decisions and the sorts of cognitive trade-offs under discussion.^8^

First, developing a skill through participation in an extra-curricular activity does not directly impair the skills that would have been developed had a different activity been selected: practicing music, for example, does not directly impair the ability to speak French. The significance of this disanalogy with cognitive trade-offs will depend upon two things. First, the extent to which the failure to develop a capacity is comparable (in an opportunity-limiting sense) to directly impairing it: if, later in life, non-developed skills can more easily be developed than impaired skills, then the child retains more options. Second, the permanency of the impairment will be highly relevant: temporary enhancement may only result in temporary impairment. If impairment to a capacity subsides, or is compensated for, then it becomes equivalent to a non-developed capacity and the (moral) distance between traditional intervention and neuro-intervention decreases.

Thus, neuroscientific evidence regarding the permanency—and extent—of cognitive costs associated with brain stimulation will be essential to determining the permissibility of parental “enhancement” decisions. It will also be crucial to know how these effects differ between one-off vs. repeated interventions, as well as whether the sought-after benefit can be achieved later in life, when the (future) adult can decide for himself or herself. Such knowledge is currently lacking. Accordingly, we highlight the need for careful consideration of these variables, and conclude that “enhancements” involving significant long-term cognitive tradeoffs should be delayed until the individual to be affected can express a considered preference (i.e., adolescence).

## Conclusion

Whilst adults are in a position to decide whether effect X is valuable enough (to them) to justify incurring impairment Y, children do not yet have the capacity or the life experience to make such trade-off decisions. They do not know what they will value when they grow up and nor do their parents. Whilst an intervention that improves X may count as an enhancement for the individual who does not care much about Y, another individual, valuing Y over X, will view the very same outcome as an impairment. In such cases—that is, cases in which the very status of an intervention’s being an (overall) enhancement vs. an impairment is controversial—the weight of considerations should shift toward delaying the intervention until the individual who will actually be affected by it has sufficient capacity to decide. The more permanent and substantial the trade-off, the more this argument has force.

The gaps Davis identifies in the literature on brain stimulation suggest that we do not currently have enough evidence to properly assess the magnitude and permanency of any trade-offs and, consequently, that the caution he recommends is indeed warranted. However, we have suggested that even when science can tell us about the effects of brain stimulation in more detail, the permissibility of parental decision-making may remain limited in some cases in which the aim is only to “enhance” an intact cognitive capacity. In contrast, the treatment of atypical cognitive abilities using brain stimulation will be permissible insofar as the stimulation is (at least) as safe and effective as existing treatments in providing an overall benefit to the child.

## Conflict of interest statement

Roi Cohen Kadosh has filed a patent entitled “Apparatus for Improving and/or Maintaining Numerical Ability” (International Application PCT/GB2011/050211).

## Footnotes

^1^Consistent with this perspective, a recent review concluded that there is at least preliminary evidence of a therapeutic potential for TMS and/or tDCS in children with conditions such as depression and autism spectrum disorder; however, it should be noted that many of the studies included in this review did not have adequate control groups, and should therefore be interpreted with care (Vicario and Nitsche, 2013).

^2^They argue that the term “non-invasive” “is inappropriate and perhaps oxymoronic, as it obscures both the possibility of side-effects from the stimulation, and the longer-term effects (both adverse and desirable) that may result from brain stimulation. [Moreover, the] tendency for the effects of [such stimulation] to spread from the target brain area to neighboring areas is in itself contrary to the definition of non-invasiveness” (p. 1). Indeed, this ability for (intended) effects of brain stimulation to have potentially adverse ramifications for other brain areas (and/or functions) is a key component of our ethical analysis.

^3^We do not suggest, of course, that there is a clear-cut, universally agreed-upon distinction between treatment and enhancement (see Maslen et al., 2014). Instead, we envision a sliding scale from interventions that are intended simply to sharpen a certain cognitive skill in a healthy child (“enhancement”) to those intended to relieve a child of pain or another burden that significantly affects his or her ability to pursue the normal range of activities that children pursue (“treatment”).

^4^Throughout this paper we invoke three overlapping considerations: the child’s “developing autonomy” (her learning to be self-governing), the child’s “future autonomy” (her prospects for pursuing the life plans that she will come to value as an adult) and the child’s “self-determination” (the freedom for her actions to be “up to her”). All three are relevant and closely related in our discussion. However, we use the term “future autonomy” more prominently, as this denotes best the concern with preserving options for the child to evaluate herself, once she has sufficient capacity to make such assessments.

^5^However, note that the permissibility of parents’ choosing the intervention would depend in part on what specific “negative side effects” might be incurred by the stimulation, as well as the magnitude of the risk. As we discuss later on, one high-risk side-effect of some kinds of brain stimulation is the diminishment of a non-targeted cognitive capacity. In this case, the persistence of symptoms due to the neurological condition would have to be worse—that is, more contrary to the child’s interests—than the cognitive trade-offs incurred by the stimulation (alongside any other negative side-effects and costs) for the stimulation to be considered permissible on the best interests test.

^6^What is assent? Although there are several different theories of assent, at its most basic, it involves agreement to or acceptance of the intervention. It is often argued that the requirements for assent are less cognitively demanding than for consent (see John et al., 2008; Waligora et al., 2014), such that individuals whose capacities to make informed judgments are still developing may nevertheless be able to meet them. In relation to assent for pediatric research, for example, Roth-Cline and Nelson suggest that the child should “understand why he or she is being asked to participate and what will be his or her experience if he or she decides to participate” (Roth-Cline and Nelson, 2013, p. 296). Precise age ranges vary, but a child’s assent is thought to be (ethically) obtainable by approximately age five or six, depending upon the specific intervention being proposed (including its risk profile, etc.), and also adjusting for the child’s individual stage of development.

^7^Therefore, a simple understanding of the immediate experience of the intervention would not be sufficient to make the enhancement intervention morally permissible. Indeed, the *acceptance* that is characteristic of child assent must be supplemented with a strong, considered *preference* in cases in which long-term trade-offs are under consideration.

^8^There will, of course, be examples of “hyperparenting” (see Sandel, 2009), which may in fact be as problematic as electing opportunity-limiting interventions involving direct intervention into a child’s brain—and for similar reasons. This will be the case particularly when the child is made to spend a considerable amount of time developing a certain skill despite her sustained dissent. Whilst parents should encourage children to try different activities, and sometimes override dissent when a child is less-than-enthusiastic on a particular occasion, a child’s long-term resistance to an extra-curricular activity renders parental force morally questionable at best, and morally impermissible at worst. This is due to the failure of such parental pressure to nurture the child’s developing autonomy and its prevention of the child’s pursuit of alternative extra-curricular options, which may become increasingly difficult to master as time goes on.
